# Treatment of Cholera in Louisville

**Published:** 1849-07

**Authors:** 


*Treatment of Cholera in Louisville.*—In the generality of cases treated by the physicians of Louisville, the treatment has been attended with the most gratifying success. The rate of mortality has been unusually small, and it has been so on account of the judicious, energetic, and discriminating character of the remedies employed. These have varied according to the circumstances we have named, as operating upon every scientific physician in making up his judgment upon each individual case. No routine system of treatment has been resorted to among the physicians of this city.

The plan usually adopted in the very early stages, is to give calomel, in doses varying from 10 grains to ℈j, combined with small portions of powdered opium, black drop, or laudanum. Sometimes the saccharum saturni is added. If there is great irritability of the stomach at this stage it is combatted best with small portions of calomel and black drop, given at short intervals. In some cases where calomel, in doses of ten grains every two hours, was given with one grain doses of opium, and where the failure to relieve was decided, we have seen the best results flow from giving calomel in three grain doses, combined with three drops of the acetated tincture of opium, every fifteen or twenty minutes. Some cases have been saved in this way that were rapidly running into collapse, under the use of larger doses given at greater intervals. We think, however, that the opium should be used with all possible moderation, and that its employment should be dispensed with as soon as it can be with safety to the patient. We prefer doing without it in all cases where it can be safely omitted. The free use of it in cholera is not attended with a very gratifying control over the disease, nor with any very flattering results in the stage of convalescence. But we consider calomel the sheet-anchor of the treatment, and feel sure, reports to the contrary notwithstanding, that no other treatment of the cholera has ever been so successful. In a conflict between the testimony of others, and the evidence of our own senses, we unhesitatingly prefer believing the latter. We have heard much vaunting of the triumphant use of other meams than mercurials, and we have seen many efforts at doing without calomel in the treatment of cholera, but we have yet to see the first evidence of a result that would tempt us to lay aside its use. That this great remedy is often used improperly, indiscreetly, and disastrously, is certainly true, but these facts are not to be considered as having any bearing upon the proper use of the medicine. It is contrary to the rules of philosophy to argue against the use of anything on account of the abuse of it. Nor are we compelled to believe all we hear or read. An ample experience in the cholera of 1832, 1833, and 1849 in Louisville, furnishes us with the most conclusive evidence of the safety and value of the remedial powers of calomel, nor do we know of anything that shakes the proof. We are not wedded to that remedy, and when we see anything that is to be compared with it, for a controlling influence over cholera, we shall cheerfully adopt it. The proximate cause of cholera—that which when present causes the disease, which when changed, changes it, and which when removed, removes it, is an inactivity of the liver, kidneys, and skin, and great irritability of the stomach and bowels. In the whole range of the Materia Medica we know of nothing that exerts the power, in this state of things, that calomel does, when combined with small portions of opium or black drop. The enlightened physician will always remember that he is using powerful means to subdue disease, not the patient, and he will cease the use of such means at the earliest moment he can, with safety to the patient. Calomel is essential in the treatment of cholera, but it is not necessary to use it in such enormous quantities as are sometimes given. In not a limited experience, we declare, that we have never seen a case of undoubted cholera recover without the administration of calomel, nor have we ever seen any disastrous consequences as the result of its use. We do not undertake to say what others have seen: we speak only of our own experience.

In the initial stages of cholera, of which we have thus far spoken, the recumbent posture, steadily maintained, is a *sine qua non*; the feet should be bathed in mustard water, and the abdomen should be covered with a mustard plaster. In some obstinate cases of diarrhea and suppression of urine, we have seen uniform good effects from the application of mustard to the spine, throughout its entire length.

But it is not often the good fortune of the physician to see a case at this early period of the developement of the disease. He is not often called in until the vomiting, diarrhea, and spasms have become very urgent. At this stage the powers of life are rapidly waning; the fluids are in swift retreat from the surface to the interior of the body; the skin is cold and clammy; the pulse is sinking; the respiration is labored; and everything denotes that collapse is approaching. In this state of things whatever is done must be done quickly and with energy.

The course found most successful here, when collapse is approaching, is first: the extensive application of sinapisms of mustard—they should be applied dry to the feet and hands, arms and legs. The mustard poultice to the spine and abdomen, is invaluable, and lumps of unslaked lime, wrapped in *wet* cloths, laid around the patient underneath the bed-clothes, have a spedier and finer effect than any other method of applying heat we have ever known. In the most of such cases there is no time to lose in waiting for hot bricks, nor are bottles of hot water very eligible. The lime wrapped in wet cloths, commences its activity immediately, and we have seen it restore the capillary circulation in cases where its return seemed utterly hopeless.

These are the first and most important methods of treatment in these desperate circumstances. The next thing to be done is to administer calomel in from ten to twenty grain doses, with black drop in doses of ten or fifteen drops, and thirty drops of camphor dissolved in chloroform. The latter is made by dissolving ʒiij of powdered camphor in ʒi of chloroform. It is rarely necessary to repeat the camphor and black drop, and we have rarely ever seen any remedy that exercised a more certain or more potent influence over congestion, than this camphor and chloroform. We have repeatedly seen this solution rouse the system from the profoundest torpor, and in which we have uniformly met with failure in the use of all other means.

After the capillary circulation is once more in healthful action, the liver must be relieved by those judicious measures which are in the hands of all intelligent practitioners. It should never be forgotten that the resources of medical science are ample for the control of cholera, and if they are properly applied, they cannot fail to triumph over this deadly distemper.

We intended to give a detail of several interesting cases that have occured in this city, but we find that we have not sufficient space for them in this department of the Journal, for this month. We shall endeavor to present them to our readers in the July number, in order to illustrate the value of the means of cure we have indicated.

We will only add, that since the report of the board of health, on the 25th, no death from cholera in the city has come to our knowledge.

May 31, 1849.—*Western Journal. Editorial.*

